# Training in Interventional Radiology: A Simulation-Based Approach

**DOI:** 10.1177/2382120520912744

**Published:** 2020-04-13

**Authors:** Indrajeet Mandal, Utkarsh Ojha

**Affiliations:** 1 John Radcliffe Hospital, Oxford University Hospitals NHS Trust, Oxford, UK; 2 Royal Lancaster Infirmary, University Hospitals of Morecambe Bay NHS Foundation Trust, Lancaster, UK

**Keywords:** Interventional radiology, training, simulation, medical technology

## Abstract

Innovations in medical technology have revolutionised both medical and surgical practice. Indeed, with such innovations, training for specific specialties has become more advanced and streamlined. However, despite these novel approaches to train students and specialist trainees, training for interventional radiology (IR) is lagging. While the reason for this lag remains contentious, one of the primary reasons for this issue may be the lack of standardisation for IR training due to a scarcity of specific guidelines for the delivery of IR procedural training. Interventional radiologists manage a vast array of conditions and perform various procedures. However, training for each procedure is largely dependent on the centre and access to a range of cases. Recently, the use of simulation technology has allowed this issue to be addressed. Simulation technology allows trainees to participate in a range of procedures regardless of their centre and availability of cases. Specialties such as cardiology and vascular surgery have already adopted simulation-based technology for trainees and have commented positively on this approach. However, simulation-based training is still lacking in the IR training pathway. Here, we evaluate why IR training can benefit from a more simulation-based approach. We further consider the cost-effectiveness of implementing simulation-based training nationally. Finally, we outline the potential pitfalls that may arise of introducing simulation-based training for IR trainees. We conclude that despite its disadvantages, simulation training will prove to be more cost-efficient and allow standardisation of IR training.

## Introduction

Interventional radiology procedures have revolutionised the field of medicine. Interventional radiologists now perform a huge range of procedures for many conditions, including haemorrhage, cancer, and vascular disease. Interventional radiology services are essential for both emergency and elective treatment, and with the development of mechanical thrombectomy, these skills will become increasingly important in the treatment of stroke. The development of minimally invasive treatments using image guidance has allowed quicker patient recovery and reduced mortality, and there is a growing need for interventional radiology procedures in the United Kingdom. Interventional radiology procedural training largely follows the traditional apprenticeship model, where a trainee scrubs with a senior on a patient case and learns by doing. However, training via this model heavily relies on the trainer and risks variation in teaching standards, as well as potentially affecting patient safety if a trainee has minimal experience before performing procedures on a real patient. Other specialties that perform image-guided interventional procedures, particularly interventional cardiology, have embraced simulation as a way of developing technique outside of the cath lab, and the curriculum for interventional cardiology lists simulation as a learning method for learning catheter skills outside of the cath lab.^1^ The Royal College of Radiologists (RCR) and British Society of Interventional Radiology (BSIR) do not mention the role of simulation in the IR training curriculum.^2^ Although the newer ‘radiology academies’ mention simulation as a method,^3^ no details are provided on its specific role in procedural training. Simulation training has the potential to help trainees learn practical skills outside the cath lab, reducing the learning curve during real cases, facilitating the learning process. However, many of the simulators that have been developed are expensive and have been criticised for low fidelity. Here, we present our viewpoint on the use of simulation for better training and finding the balance between effective simulation training and being cost-effective.

## Usage and Outcomes of Simulation Training in Other Catheter-Based Intervention

Other medical specialties have shifted towards simulation-based training and have reported successful outcomes in educating their trainees. In cardiology, gaining arterial access is a key procedure required for cardiac catheterisation. Gurm et al^4^ compared outcomes among trainee cardiologists in gaining femoral access during cardiac catheterisation. The group found that those who had undertaken simulation training had reduced patient complications and reduced time in achieving 5 successful unassisted femoral artery access than those who had undertaken the standard, non-simulation-based training. Moreover, studies^5,6^ have also reported reduced procedural times in cardiac angiography in those who have undertaken simulation-based training. Reduction in procedural time minimises risk of intraoperative complications and also demonstrates the trainees’ procedural skills. These findings are further supported by Bagai and colleagues^7^ who evaluated whether simulation training affects trainees’ ability to perform cardiac catheterisation in real patients. The authors randomised 27 cardiology trainees into 2 groups: one group underwent simulation-based training and the control group took standard apprenticeship alone. The study showed that those trainees who undertook simulation-based training demonstrated a significantly improved technical ability than the control group.

In the United Kingdom, simulation-based training is regarded as an important training tool among vascular surgery programme directors.^8^ In 2017, Robinson et al^9^ investigated the impact of a vascular surgical skills and simulation course on the procedural knowledge and self-rated competence of vascular surgery trainees. In this study, candidates took a 1.5-day course entailing open and endovascular procedures on high-fidelity simulators and cadavers. The group reported that this simulation-based workshop significantly increased the self-rated competence among the trainees. Furthermore, in a nationwide study^10^ across the United States conducted by The Association of Program Directors in Vascular Surgery Committee on Education and Simulation reported that operative experience and confidence is increased among those trainees who have access to simulation-based training.

Endovascular procedures have become a critical technique for neurosurgeons, especially when performing cerebral angiography, thrombolytic therapy, endovascular coiling carotid artery stenting, and angioplasty. This discipline is heavily dependent on imaging. Indeed, in 2010, the Congress of Neurological Surgeons (CNS) established a committee termed ‘CNS Simulation Committee’. The aim of this committee was to maximise resident education and improve patient outcome by creating simulation-based training. Fargen et al,^11^ developed an endovascular course to assess the outcomes among neurosurgery trainees who underwent simulation training. In this study, 37 participants were assessed on their ability to perform diagnostic angiogram using simulators. The study found these participants showed significant improvements in their overall performance following simulation training. In 2013, Choudhury and colleagues^12^ developed a conceptual training framework for use on virtual reality neurosurgical simulator. The authors identified appropriate skills from the neurosurgical oncology curricula including ventriculostomy, endoscopic nasal navigation, tumour debulking, haemostasis, and microdissection. These tasks were elaborated onto training modules and were deemed as useful for neurosurgical skills training by subject matter experts. Following the development of similar conceptual training frameworks, engineers partnered with neurosurgeons of the National Research Council of Canada to establish the NeuroTouch neurosurgical simulator. This simulator offers trainees several scenarios based on real-life cases including endonasal pituitary surgery and transsphenoidal surgery.^13^ Taken together, it is evident that several medical and surgical specialties have trialled incorporating simulation-based training in their curricula and have shown positive outcomes in improving trainees’ competency and skill as well as improving patient safety and care. A summary of simulator use in other specialties is provided in Table 1.

**Table 1.** Status of simulation training in other specialties.

Field	Curriculum statement on simulation	Procedures	Existing literature summary	Key papers
Vascular surgery	Required part of specialty induction for some skills Recommended for some skills	Peripheral artery stenting Renal artery stenting Carotid artery stenting	Simulation improves performance in novices and advanced trainees. Benefit is higher for novices. Expert feedback further augments simulation training. Simulation training is still beneficial post-night shift. Simulation is an effective warm up tool prior to performing the procedure on a patient.	Dayal et al, 2004^14^ Hsu et al, 2004^15^ Aggarwal et al, 2006^16^ Dawson et al, 2006^17^ Chaer et al, 2006^18^ Neequaye et al, 2007^19^ Passman et al, 2007^20^ Boyle et al, 2011^21^ Naughton et al, 2011^22^ Willaert et al, 2011^23^
Interventional cardiology	Recommended as a learning method for practical skills outside catheter lab	Cardiac catheterisation	Simulation improves performance in novices and advanced trainees. Benefit is higher for novices.	Patel et al, 2006^24^ De Ponti et al, 2011^25^ Bagai et al^7^ Voelker et al, 2016^26^ Schimmel et al^6^ Casey et al^5^
Neurosurgery	Strongly recommended for competencies at each stage of training	Angiography	Simulation improves performance in novices and advanced trainees.	Fargen et al, 2012^27^ Spiotta et al, 2013^28^ Fargen et al, 2013^11^

## Benefits of Simulation Training

The use of simulators as an adjunct to training offers a huge number of potential benefits. The main benefit is that trainees reduce the learning curve for new procedures.^29,30^ Using simulators, trainees can learn the basic catheter skills outside the cath lab, which allows them to focus their attention on the more advanced aspects of the procedure in a real case. This would overall reduce the number of procedures required to achieve proficiency. This would provide large benefits for patient safety, as trainees would have had the chance to hone the basics when first performing the procedure, resulting in reduced radiation doses and sedation times. In addition, by reducing the number of procedures required, trainees would be able to get exposure to procedures that do not have a high caseload at their institution. This allows standardisation of training across the country and ensures that all trainees are able to learn certain procedures. Simulators are also accessible at any time, which gives the trainee more flexibility as opposed to learning whenever cases occur. Another advantage of simulators is that it can provide early exposure to IR procedures. This is particularly important for a recruitment perspective, and early exposure has shown to increase interest in IR for medical students. In 2011, a meta-analysis^31^ examined studies over 20 years regarding the skill acquisition from simulation-based training compared with traditional training. In this analysis, the authors identified 3742 articles, of which 14 met the inclusion criteria. The studied showed that the overall effect size for the 14 studies comparing the effectiveness of simulation-based training compared with traditional clinical training was 0.71 (95% confidence interval: 0.65-0.76; *P* < .001). Although the sample size of the 14 studies was relatively small, the duration over which these studies were analysed shows that there is a consistent positive impact on education following simulation training. However, it can be argued that the cost-effectiveness and other logistics were not factored when comparing the 2 interventions.

Simulators exist in multiple forms, but the most common forms are virtual reality simulators and phantom simulators. Table 2 shows the commercially available endovascular simulators currently on the market.

**Table 2.** Summary of currently available simulators.

Simulator	Manufacturer	Type	Available modules	Link
VIST	Mentice	Endovascular VR	Neuro: Carotid, thrombectomy, coiling, stroke intervention Cardiac: PCI, EP, TAVI, ASD/PFO, LAAO Aortic: EVAR, TEVAR Embolotherapy: PAE, UAE, TACE Peripheral: renal, iliac/SFA, below knee Other: Trauma	www.mentice.com/simulators
Angio Mentor	3D Systems	Endovascular VR	Neuro: Carotid, cerebral, stroke intervention Cardiac: coronary, EP, trans-septal puncture, AF ablation, ASD, TAVI, LAA closure Aortic: EVAR, TEVAR Embolotherapy: peripheral, PAE, venous Peripheral: Renal, iliac, SFA, lower extremity, below knee, atherectomy Other: Trauma	www.simbionix.com/simulators/angio-mentor
ORCAMP	ORZONE	Endovascular VR	Neurointervention and stroke Cardiology Vascular surgery Interventional radiology	www.orzone.com/team-training
EVE	FAIN Biomedical	Endovascular VR	Neuro: Carotid, aneurysm Cardiac: PCI Peripheral: hepatic and renal arterial intervention	www.fain-biomedical.com/eve01/
CathLabVR	CAE Healthcare	Endovascular VR	Cardiac: PCI, TAVI, EP Neuro: Carotid	www.caehealthcare.com/surgical-simulation/cathlabvr/

## Limitations of Simulation Training

Simulation training in its current form is not without its problems. The biggest issue with the current simulators is fidelity. Many of the current simulators do not accurately reflect the actual procedure, which limits its effectiveness. This makes purchasing a simulator a large financial commitment that may not achieve the desired result. A specific challenge for IR is the issue of simulating many different procedures in different body systems. The range of procedures is much greater in IR compared with other specialties that use simulation, and a simulator that truly acts as an adjunct to IR training would ideally be able to offer simulation for a wide range of procedures. Financial barriers exist to the widespread use of simulators. IR residency programmes should work with local cardiology, vascular surgery, and neurosurgery programmes to share the financial burden of purchasing simulators and provide benefits to all trainees. A solution could be to hold a common endovascular skills course for all trainees in these 4 specialties at the start of training. In addition, there is currently not enough objective evidence for simulation training specifically in IR. Further evidence is required to demonstrate that simulation training is beneficial for trainee-focused outcomes, procedure-based variables, and patient outcomes.

## Moving Forward: Questions to Consider

The question of when simulation training should be offered still remains. Specialty recommendations in the United Kingdom currently vary. Vascular surgery includes mandatory simulation training at induction, as well as recommending continued use of simulation throughout training. However, neurosurgery and interventional cardiology recommend supplementing skills with simulation throughout training without a formal requirement. Evidence suggests that there is a benefit of simulation training for both newcomers and advanced practitioners.^32^ Given the range of procedures required in IR residency, we recommend that there should be a required simulation training curriculum at the beginning of residency to reduce the learning curve during residency and facilitate acquisition of catheter-based skills. Further simulation should be recommended throughout residency as an adjunct to improve specific aspects of a particular procedure that an individual trainee may require further practice on.

The length of an ideal simulation training programme is yet to be determined. Previous studies have varied, and this ranges from a single 30-minute session to a 2-day intensive course. Although simulation has shown benefits for both novice and experienced practitioners, there is evidence of diminishing returns as a trainee gains proficiency in the procedure. Therefore, there needs to be a balance of offering the highest benefit for trainees vs increasing time demands on an already busy residency schedule. Outcome measures of simulation training are also an important issue. Previous studies have used 3 main metrics – simulator metrics, evaluation by an experienced physician, and questionnaire responses from subjects. However, future work should evaluate the success of training on patient-based outcomes and assess the impact of simulation on patient outcomes.

The expanding range of procedures in IR has meant a high degree of technical ability is critical to a successful resident. With this in mind, the dilemma of whether we should use performance metrics in selection for residency has raised. We would recommend against using simulator-based assessment for residency selection. The evidence shows that inexperienced practitioners improve with time. Therefore, pre-residency success on such a test would largely be determined by whether a student has had exposure to the simulator or not.

## Concluding Remarks

Interventional radiology is a rapidly expanding specialty. Indeed, simulation-based training has become a novel and exciting technique to educate trainees in interventional procedures. While other medical and surgical specialties have adopted simulation training to teach catheter-based interventions, interventional radiology still lacks such innovative methods for training. We envisage that simulation-based training can help shape the interventional radiology training pathway to become more standardised. Simulation training can provide access to a range of real-life cases which may not be as easily accessible across all centres. This method of training can better equip trainees with the correct skill set and self-confidence when performing procedures which can ultimately lead to better and safer patient care.

## References

[1] Joint Royal Colleges of Physicians Training Board. Specialty Training Curriculum for Cardiology. Updated 2016. https://www.jrcptb.org.uk/sites/default/files/2010%20Cardiology%20Curriculum%20%28amendments%202016%29.pdf.

[2] Royal College of Radiologists. Sub-Specialty Training Curriculum for Interventional Radiology. Updated 2016. https://www.rcr.ac.uk/clinical-radiology/specialty-training/curriculum/interventional-radiology-curriculum.

[3] Royal College of Radiologists. Radiology Training 2016-2026: A Vision and a Solution. Updated 2016. https://www.rcr.ac.uk/sites/default/files/vision_for_training.pdf.

[4] Gurm HS Sanz-Guerrero J Johnson DD, et al Using simulation for teaching femoral arterial access: a multicentric collaboration. Catheter Cardiovasc Interv. 2016;87:376-380. 26489781 10.1002/ccd.26256

[5] Casey DB Stewart D Vidovich MI. Diagnostic coronary angiography: initial results of a simulation program. Cardiovasc Revasc Med. 2016;17:102-105. 26811001 10.1016/j.carrev.2015.12.010

[6] Schimmel DR Sweis R Cohen ER Davidson C Wayne DB. Targeting clinical outcomes: endovascular simulation improves diagnostic coronary angiography skills. Catheter Cardiovasc Interv. 2016;87:383-388. 26198625 10.1002/ccd.26089

[7] Bagai A O’Brien S Al Lawati H, et al Mentored simulation training improves procedural skills in cardiac catheterization: a randomized, controlled pilot study. Circ Cardiovasc Interv. 2012;5:672-679. 23048053 10.1161/CIRCINTERVENTIONS.112.970772

[8] Ghatan CE Kuo WT Hofmann LV Kothary N. Making the case for early medical student education in interventional radiology: a survey of 2nd-year students in a single U.S. institution. J Vasc Interv Radiol. 2010;21:549-553. 20189831 10.1016/j.jvir.2009.12.397

[9] Robinson WP Doucet DR Simons JP, et al An intensive vascular surgical skills and simulation course for vascular trainees improves procedural knowledge and self-rated procedural competence. J Vasc Surg. 2017;65:907.e3-915.e3. 10.1016/j.jvs.2016.12.065 28236930

[10] Duran C Bismuth J Mitchell E. A nationwide survey of vascular surgery trainees reveals trends in operative experience, confidence, and attitudes about simulation. J Vasc Surg. 2013;58:524-528. 23541545 10.1016/j.jvs.2012.12.072

[11] Fargen KM Arthur AS Bendok BR, et al Experience with a simulator-based angiography course for neurosurgical residents: beyond a pilot program. Neurosurgery. 2013;73:46-50. 24051882 10.1227/NEU.0000000000000059

[12] Choudhury N Gelinas-Phaneuf N Delorme S Del Maestro R. Fundamentals of neurosurgery: virtual reality tasks for training and evaluation of technical skills. World Neurosurg. 2013;80:e9-e19. 23178917 10.1016/j.wneu.2012.08.022

[13] Delorme S Laroche D Diraddo RF Del Maestro R. NeuroTouch: a physics-based virtual simulator for cranial microneurosurgery training. Neurosurgery. 2012;71:32-42. 22233921 10.1227/NEU.0b013e318249c744

[14] Dayal R Faries PL Lin SC Bernheim J Hollenbeck S Derubertis B, et al Computer simulation as a component of catheter-based training. J Vasc Surg. 2004 12 1;40(6):1112–1117. 15622364 10.1016/j.jvs.2004.09.028

[15] Hsu JH Younan D Pandalai S Gillespie BT Jain RA Schippert DW, et al Use of computer simulation for determining endovascular skill levels in a carotid stenting model. J Vasc Surg. 2004 12;40(6):1118–1125. 15622365 10.1016/j.jvs.2004.08.026

[16] Aggarwal R Black SA Hance JR Darzi A Cheshire NJW Virtual Reality Simulation Training can Improve Inexperienced Surgeons’ Endovascular Skills. Eur J Vasc Endovasc Surg. 2006 6;31(6):588–593. 16387517 10.1016/j.ejvs.2005.11.009

[17] Dawson DL. Training in carotid artery stenting: Do carotid simulation systems really help? Vascular. 2006 9;14(5):256–263. 17038295 10.2310/6670.2006.00045

[18] Chaer RA DeRubertis BG Lin SC Bush HL Karwowski JK Birk D, et al Simulation improves resident performance in catheter-based intervention: Results of a randomized, controlled study. Ann Surg. 2006 9;244(3):343–9. 10.1097/01.sla.0000234932.88487.75 PMC1856536 16926560

[19] Neequaye SK Aggarwal R Brightwell R Van Herzeele I Darzi A Cheshire NJW Identification of Skills Common to Renal and Iliac Endovascular Procedures Performed on a Virtual Reality Simulator. J Vasc Surg. 2007 5 1;45(5):1088. 10.1016/j.ejvs.2006.12.022 17291792

[20] Passman MA Fleser PS Dattilo JB Guzman RJ Naslund TC. Should simulator-based endovascular training be integrated into general surgery residency programs? Am J Surg. 2007 8;194(2):212–9. 10.1016/j.amjsurg.2006.11.029 17618806

[21] Boyle E O’Keeffe DA Naughton PA Hill ADK McDonnell CO Moneley D. The importance of expert feedback during endovascular simulator training. J Vasc Surg. 2011 7;54(1):240-248.e1.22. 21636241 10.1016/j.jvs.2011.01.058

[22] Naughton PA Aggarwal R Wang TT Van Herzeele I Keeling AN Darzi AW, et al Skills training after night shift work enables acquisition of endovascular technical skills on a virtual reality simulator. J Vasc Surg. 2011 3 1;53(3): 858–66. 10.1016/j.jvs.2010.08.016 20952142

[23] Willaert WIM Aggarwal R Van Herzeele I O’Donoghue K Gaines PA Darzi AW, et al Patient-specific endovascular simulation influences interventionalists performing carotid artery stenting procedures. Eur J Vasc Endovasc Surg. 2011 4;41(4):492–500. 21276738 10.1016/j.ejvs.2010.12.013

[24] Patel AD Gallagher AG Nicholson WJ Cates CU. Learning Curves and Reliability Measures for Virtual Reality Simulation in the Performance Assessment of Carotid Angiography. J Am Coll Cardiol. 2006 5 2;47(9):1796–802. 10.1016/j.jacc.2005.12.053 16682303

[25] De Ponti R Marazzi R Ghiringhelli S Salerno-Uriarte JA Calkins H Cheng A. Superiority of Simulator-Based Training Compared With Conventional Training Methodologies in the Performance of Transseptal Catheterization. J Am Coll Cardiol. 2011 7 19;58(4):359–63. 10.1016/j.jacc.2011.02.063 21757112

[26] Voelker W Petri N Tönissen C Störk S Birkemeyer R Kaiser E, et al Does Simulation-Based Training Improve Procedural Skills of Beginners in Interventional Cardiology? - A Stratified Randomized Study. J Interv Cardiol. 2016 2 1;29(1):75–82. 26671629 10.1111/joic.12257

[27] Fargen KM Siddiqui AH Veznedaroglu E Turner RD Ringer AJ Mocco J. Simulator based angiography education in neurosurgery: Results of a pilot educational program. J Neurointerv Surg. 2012 11;4(6):438–41. 10.1136/neurintsurg-2011-010128 22015637

[28] Spiotta AM Rasmussen PA Masaryk TJ Benzel EC Schlenk R. Simulated diagnostic cerebral angiography in neurosurgical training: A pilot program. J Neurointerv Surg. 2013 7;5(4):376–81. 10.1136/neurintsurg-2012-010319 22576472

[29] Klass D Tam M Cockburn J Williams S. Training on a vascular interventional simulator: an observational study. Eur Radiol. 2008;18:2874-2878. 18618118 10.1007/s00330-008-1090-y

[30] Narra P Kuban J Grandpre LE Singh J Barrero J Norbash A. Videoscopic phantom-based angiographic simulation: effect of brief angiographic simulator practice on vessel cannulation times. J Vasc Interv Radiol. 2009;20:1215-1223. 19729133 10.1016/j.jvir.2009.06.006

[31] McGaghie WC Issenberg SB Cohen ER Barsuk JH Wayne DB. Does simulation-based medical education with deliberate practice yield better results than traditional clinical education? A meta-analytic comparative review of the evidence. Acad Med. 2011;86:706-711. 21512370 10.1097/ACM.0b013e318217e119 PMC3102783

[32] Coates PJB Zealley IA Chakraverty S Endovascular simulator is of benefit in the acquisition of basic skills by novice operators. J Vasc Interv Radiol. 2010;21:130-134. 19931470 10.1016/j.jvir.2009.09.013

